# Foreign Summary

**Published:** 1839-12-28

**Authors:** 


### FOREIGN SUMMARY.

### Velpeau's Clinical Lectures on Ophthalmia, No. XIII.

*Terminations of iritis.—Prognosis.—Treatment of acute iritis.—General treatment.*

*Terminations of iritis.*—Iritis may terminate in several ways; by resolution; by the formation of false membranes, or adhesion of the iris to the surrounding tissues; by transformation into some other disease; or by suppuration.

Resolution is the most frequent and at the same time the most desirable termination of iritis. When it takes place the inflammatory symptoms gradually become less acute. The redness of the conjunctiva, as also that of the radiated sclerotic zone, diminishes, and at last entirely disappears. The iris appears less tumefied, and again assumes the smooth appearance which it presents in the healthy state. The external and internal zones return in a great measure to their natural colour, the pupil recovers its mobility and its regularity of form, and the cornea, as also the aqueous humour, again becomes transparent. The clarification of the aqueous humour is, indeed, one of the first symptoms which announce the resolution of the inflammation, and when it takes place we are justified in making a favourable prognosis. The false membranes, the flakes of coagulable lymph, are gradually absorbed, and the collections of blood or lymph which occur in the tissue of the iris itself, and of which I have already spoken, become flattened, circumscribed, and are finally resolved.

The resolution is not, however, always as complete after acute inflammation of the iris, as I now describe it to be. The abscesses may leave indelible marks, the coagulable lymph which is effused in the pupil may not be wholly absorbed, and the iris may not entirely recover its mobility. Indeed, when adhesions have been formed, the resolution is seldom perfect.

There are two forms of adhesion of the iris. Adhesion may exist between the iris and the adjoining tissues, thus giving rise to the synechia of pathologists, or it may exist between the fibres of the iris itself. The iris is formed by a great number of circular and radiated fibres, which are supposed by some physiologists to be muscular, but in my opinion their views are erroneous. Whatever may be their nature, these fibres, which act separately and are naturally very moveable, may become united with one another in such a manner as to lose partly or even entirely their usual mobility. I cannot give you a better idea of the nature of the union that takes place than by comparing it to that which would occur between the fingers of the hand, were they kept in close approximation when violently inflamed. You may not all feel inclined to recognize the existence of this species of adhesion. But on consideration you will find that it is only by allowing that adhesion does take place between the fibres of the iris, that we can satisfactorily explain the coarctations of the pupil, coarctations which we often observe even when the iris has contracted no adhesion whatever with the adjoining tissues. We have at the present time in our male wards a case which will illustrate this form of adhesion, the coarctation of the pupil being carried to a great extent, without there being any apparent connexion between the iris and the organs placed posteriorly. The patient, a man of about fifty years of age, was affected with acute iritis of the left eye, several years ago, and the pupil was so contracted when he entered the hospital, this day fortnight, that it would scarcely have admitted the head of a large pin. Under the influence of belladonna, the pupil has become slightly enlarged, and has assumed a triangular form, something similar to an ace of clubs. The other eye is at the present time the seat of chronic inflammation, and it is for the affection of this eye that he entered the hospital. The pupil is irregular, and presents a margin prominent in some parts, slightly depressed in others. Those portions of the pupil which are depressed seem as if they were drawn back by something, whilst those which are prominent are apparently quite free. This case alone will enable you to form a very correct idea of the various modifications which adhesion of the iris may present.

The adhesion which takes place between the iris and the adjoining tissues also offers two forms, which I have already briefly described to you under the names of anterior and posterior synechia. In anterior synechia the iris may adhere to the cornea either by its entire papillary circumference, or by a few points only. This kind of adhesion is rare, and when it exists, the size of the anterior chamber appears much diminished. Posterior synechia is much more frequently met with, nor can we be surprised that this should be the case, when we consider that the distance which separates the iris from the crystalline lens is extremely slight. When the iris is inflamed it becomes tumefied, and the aqueous humour in the anterior chamber at the same time increasing, it is pushed backwards towards the lens. If the inflammation does not subside, effusion of lymph takes place, as we have already seen; the aqueous humour becomes troubled, and small filaments soon form, which, attaching themselves to the posterior surface of the iris, and to the anterior surface of the crystalline lens, constitute a connexion between the two organs. This form of adhesion may be easily recognized by rapidly raising the eyelid, previously closed for a few seconds, and then examining the eye. The pupil dilates when the eyelid is closed, but unequally, so as to assume every possible variety of shape. In some instances the adhesion takes place in another manner; the pupillary margin is connected with the crystalline lens by small filaments like hairs, a quarter of a line or half a line in length, which, tying it down as it were, occasion small triangular depressions wherever they exist. In all the forms of adhesion which I have described the functions of vision are more or less impaired.

Sometimes, when the iris has partly recovered its mobility, we see at the bottom of the pupil, near its circumference, a radiated ring. This ring is of a blackish colour, and may be compared to the sclerotic zone, of which I have so often spoken. When it is present the sight is always more or less disordered. Its existence is a proof that the inflammation has occupied the parenchyma of the iris, that pigmentum has been secreted in great abundance, and that the pupil, having rested on the anterior surface of the crystalline lens, has left this trace of its passage. Great attention has been paid to this phenomenon by German pathologists, who, to explain it, have invented a black radiated cataract. Indeed, the discussion on this subject has been rather warm, some asserting that the zone was formed by vessels, others that it was formed by pigmentum. The black radiated ring in question may be easily produced in the dead subject by depressing the cornea—a fact which shows at once that the explanation I have given you is the true one.

After acute inflammation of the iris the resolution is sometimes so imperfect as to allow the formation of false cataract or of opaque membranes, which may constitute a complete obstacle to vision. I have often seen the malady thus terminate.

Iritis may disappear under the influence of the inflammation of the eye. Thus I have several times seen it give way when the cornea or the conjunctiva have become seriously affected.

Sometimes iritis terminates by suppuration, a circumstance much to be dreaded, from the disastrous consequences by which it is followed, with regard to the functions of the organ. When the iritis is extremely intense the whole interior of the eye may be attacked by phlegmonous or purulent inflammation; this general inflammation of the eye is called *ophthalmitis* by modern authors.

*Prognosis.*—From the description I have given of iritis, it is evidently a serious disease, not as regards the life of the patient, which is seldom in danger, but as regards the functions of the affected organ, which are often impaired even when the disease is cured. When, however, the treatment is begun sufficiently early, iritis frequently gives way, especially if there is no previous opacity of the cornea, and if the inflammation has not been cured by local injury.

### *Treatment of Acute Iritis.*

Before we enter at length into the examination of the various remedial agents which are directed against iritis, I must remind you that a distinction should be made between primitive and secondary iritis; that is, between the inflammatory affection which commences by the iris, and that which is merely a consequence of the inflammation of some other organ. This distinction is important, as secondary iritis will often disappear when the affection by which it is caused has given way, and is often much less difficult to cure than primitive iritis. Local remedies also sometimes prove successful in secondary iritis, whereas they have little or no influence over the primitive form of inflammation.

The remedies which have been employed in the treatment of iritis are quite as numerous as those which have been used against keratitis, nearly every possible plan of treatment, both general and local, having been alternately tried, extolled, and rejected. I have myself tried most of the remedial agents which have been directed against iritis at various epochs, and will now lay before you the result of my experience. In doing so, I shall follow the same plan as when treating of keratitis; that is, I shall first speak of the general, and then of the local treatment of the disease. As I have said nothing hitherto of the pretended specific forms of iritis, I shall not now allude to the treatment which they may require.

*General treatment.*—General remedies, as might be expected, from the situation of the iris, occupy the first place in the treatment of iritis. They may be divided into three classes—evacuants, alteratives, and resolutives.

At the head of the first class may be placed *blood-letting*, both general and local. Blood-letting is certainly the most powerful agent that can be directed against the disease, and, at the same time, the one the utility of which is the most universally recognised. But, although practitioners are unanimous in acknowledging the efficacy of the remedy, they are far from agreeing as to the manner in which it should be employed. Some prefer general, some local bleeding; some bleed copiously at several days interval, others bleed less freely, but repeat the operation oftener; whilst others, again, extol the plan of bleeding *coup sur coup*. Before we examine the comparative efficacy of these various methods, I must remind you that general bleeding may sometimes be contra-indicated by the individual state of the patient. Thus, it cannot be resorted to, or at least carried to any great extent, with patients of a lymphatic or scrofulous habit of body, with those whose constitution has been weakened or deteriorated by disease, or by any other cause, or with those who appear to be in a state of anemia. When these contra-indications do not exist, general bleeding may be resorted to without fear. I do not, however, in any case, approve of the abstraction of a very large quantity of blood at the commencement of the disease, a plan recommended by some surgeons. Thus, Saunders advises the patient to be bled as soon as possible to the extent of two, three, and even four pounds. Such a mode of treatment is scarcely ever adopted in France. The loss of so large a quantity of blood may sometimes be useful, it is true, but it is, generally speaking, attended with very serious disadvantage. The patient is very frequently reduced to such a state of anemia and weakness, that effusion may take place in the serous cavities, or, what is more serious, the inflammation gradually returns as the strength improves, and that when his extreme weakness precludes our having recourse to the remedy we have already employed.

When the patient is strong and robust, I often bleed *coup sur coup*, that is, night and morning for several consecutive days, and, at the same time, apply leeches behind the ears in the middle of the day; but I do not make it a general rule to act in this manner. Sometimes I bleed with the lancet one day, and only apply leeches the following morning; indeed, I am guided by circumstances, and the individual state of the person I am treating. The inflammation is always favourably modified by this plan of treatment, sometimes, even, it is entirely subdued; the vascularization of the sclerotica, as also that of the conjunctiva, disappearing, and the cornea recovering its usual transparency. In some cases, however, bleeding alone will not completely subdue the disease.

Some practitioners advise blood to be taken from the foot, sooner than from the arm, because they think that blood-letting thus practised, exercises a derivative action. As, however, I cannot understand why the effect of venesection performed on the veins of the foot, should be more derivative than when performed on those of the arm, I shall not say anything further on the subject.

Local blood-letting is often associated with general blood-letting, but it is also frequently resorted to alone, when the latter mode of depletion is inapplicable, owing to the state of anemia in which we find the patient. In these cases the local abstraction of blood will produce the same effect on the inflamed membrane, and that without much loss to the economy. When leeches are employed, they may be applied either on the mastoid region, on the temples, or round the orbits. My own experience has not shown me that they are more useful in one region than in another; nor do I think that the question can easily be solved, as the efficacy of leeches is not, in the majority of cases, sufficiently evident to enable us to form a correct estimate of the benefit that has been derived from their use. In my opinion, therefore, it is of but little importance which of the regions I have named is selected, unless it be in private practice, when it is as well not to place them round the orbit, as the swelling of the eyelids to which they give rise often alarms the patient and his family. The tumefaction being merely due to serous or sanguineous infiltration, and nearly always disappearing in the course of two or three days, there is no real foundation for the fears which are entertained. I am, indeed, inclined to believe that, acting as a revulsive, it may favour the resolution of the malady. It is, nevertheless, better for the surgeon not to have recourse to a plan of treatment which may create a disagreeable impression on the mind of his patient. Leeches may also be placed, as in keratitis, on the internal surface of the eyelids, where two or three will often exercise as great an influence over the disease as fifteen or twenty applied on another region. But this is a practice which I only follow when I wish to act on the inflammatory affection of the iris, with the loss to the system of as little blood as possible.

Cupping, which is preferred by some practitioners to every other kind of local depletion, may be resorted to under the same circumstances as leeches, the indication for the one being also the indication for the other. Many surgeons think that advantage is to be derived from cupping in one region sooner than in another, but I have not, in my own practice, found this to be the case.

When the inflammation persists, after general and local blood-letting have been employed, other remedies must be tried, the most important of which are purgatives.

*Purgatives* have always been much employed in the treatment of acute iritis. You know I look upon them as irritants, which act by depriving the economy of a certain proportion of its elements. Weller extols the purgatives that are in general use, such as senna, jalap, gamboge, &c. but without appearing to entertain any peculiar theoretical ideas respecting their mode of action. Other surgeons, attributing to this class of therapeutic agents specific properties, employ the drastic purgatives, such as aloes, scammony, &c. in frequently repeated doses, with a view to irritate the intestinal canal. English practitioners, guided by the theoretical ideas which they profess, make frequent use of purgatives. In France, a short time ago, purgatives were generally accounted irritants capable of inflaming the intestinal canal, unless administered with the greatest precaution. With our friends on the other side of the channel, no such fears existing, they are constantly resorted to: calomel, either alone or associated with some other substance, is the purgative they most frequently employ. Nor can we be surprised at this, when we recollect that calomel is considered by them, not only to be an excellent antiphlogistic, but also to possess properties peculiar to itself, and is, consequently, generally introduced into their prescriptions when they consider purgatives indicated.

Calomel is employed in England as a purgative, with a view to produce mercurial action, and as an alterative. When given as a purgative, the dose is from two to six grains, repeated for several consecutive days. When given in order to produce salivation, ten, fifteen, or twenty grains are administered every day in divided doses; and lastly, when it is thought desirable to produce an alterative effect, it is employed in small doses, combined with opium or hyoscyamus, two or three times a day. Calomel has been used in the same manner in France; indeed, some of our countrymen have even gone further than the English surgeons. A medical gentleman of Avignon, for instance, having made a journey to England, became convinced that calomel was a heroic remedy against acute iritis, and determined, on his return, to give it a trial. This he did in the hospital of Avignon, where he gave, with great success, it appears, as much as twenty and thirty grains of calomel daily to his patients. I have myself given every species of purgative, and that in every possible manner, and have come to the following conclusions:—Ordinary purgatives, whether they act as evacuants or as irritants, have never appeared to me to possess much efficacy in the treatment of iritis. I have never seen the inflammation disappear in such a manner as to leave me the conviction that it was to the purgatives I had administered that its disappearance was due. I have very frequently given calomel, both as a purgative, as a mercurial agent, and as an alterative, and I must say that I have sometimes seen the iritis disappear when the economy was deeply disturbed by its action. Thus I have seen the decrease and final disappearance of the inflammation coincide with salivation, but I am not prepared to say whether this was the result of the medication I was employing, or whether it was mere coincidence. We had, a few days since, in our female ward, a woman labouring under iritis, accompanied by chronic keratitis. Blood-letting and topical remedies having proved unavailing, I gave her ten or twelve grains of calomel every day, for five successive days. The calomel caused violent purging, as also salivation, and the intensity of the iritis was, at the same time, much mitigated. This at first appears conclusive as regards the efficacy of calomel; but if we scrutinize narrowly the facts of the case, we shall find that we are not in reality authorized to draw from it such an inference. While she was taking the calomel, a blister was placed on the calf of each leg, and the application of one of these blisters was followed by severe phlegmonous erysipelas. We cannot, therefore, but ask whether the amelioration which took place is to be attributed to the salivation, or to the revulsive action set upon the leg. But this is a question which I do not feel able to answer, as I have seen in many other instances salivation productive of no benefit whatever. In fine, I believe that calomel, given in large doses, may prove useful in the treatment of acute iritis; but, on the other hand, I doubt whether the accidents to which the presence of this substance in the economy often gives rise, are not of such a nature as to more than counterbalance the benefit which may be derived from it. It is well known that salivation is sometimes followed by a form of diarrhœa which is extremely difficult to cure, as it depends on a serious lesion of the intestinal canal. In several cases of this kind which I have been able to examine after death, I have found the mucous membrane of that organ tumefied, of a grayish colour, and disorganized to a surprising extent. Knowing, therefore, that the use of calomel exposes his patient to such an affection, a surgeon must have extreme confidence in its efficacy as a remedy against the disease to employ it in every case—a confidence indeed which I am very far from possessing. I often give, however, four, eight, or ten grains, for the first day or two of the treatment.

Tincture of colchicum and oil of turpentine have been lauded by some practitioners as extremely efficacious remedies. Tincture of colchicum has been more especially recommended by M. Carron du Villards, and that principally against the specific forms of ophthalmia. I have often employed this preparation, but do not remember ever having seen the patients on whom I tried it derive much benefit from its use. The action of tincture of colchicum is in my opinion similar to that of other purgatives, and as it is a purgative on which no reliance can be placed, I dot think it will ever be much used in the treatment of iritis. Some English surgeons seem to look upon oil of turpentine as a remedy endowed with extraordinary properties. Thus Mr. Carmichael says he has used it with surprising success in iritis. He gives two, four, six, or even eight drachms, in the twenty-four hours. Messrs. Guthrie, Riggs, Mackenzie, and others, have also published cases which would tend to prove that oil of turpentine is a valuable therapeutic agent in the treatment of iritis. My own experience of the remedy is too slight to enable me to say much on the subject, but on examining the statements to which I have just alluded, I do not find that they are at all conclusive. The patients whose cases are related had all been affected three weeks, a month, or even two months, previous to being treated, and were then either cured in ten or fifteen days, or otherwise the eye became disorganized. But this is what we see continually when recourse is had to those remedies only which are usually employed. The oil of turpentine is, like the oil of coloquintida, an acrid irritating purgative, and its use is not unattended with danger to the intestinal canal. in none of the cases which have hitherto been published do I see any thing which can warrant our preferring it either to calomel or to the other purgatives which are generally used. It is, moreover, a most detestable remedy to take, and why should we choose it, when there are so many of the same nature more agreeable to the patient, and to those who surround him, and also more efficacious?

In addition to blood-letting and purgatives, there are many other general remedies which have been lauded as specifics against iritis. Such are antimonials, the golden sulphuret of antimony, the sulphur, iodine, sudorifics, &c.

I have frequently given tartarised antimony, either diluted or in sorian doses, but have never seen it exercise much influence over the course of the disease. Sometimes, however, when the iritis was already in a great measure subdued, it has appeared to accelerate the resolution of the inflammation. With regard to the golden sulphuret of antimony, it may be classed along with the nitrate of bismuth, which, although extolled for some time as an extremely efficacious remedy, is now looked upon as an inert powder. The same cannot altogether be said of sulphur and iodine. Yet I have given sulphur in doses varying from fifteen to twenty grains, without obtaining any satisfactory results. The utility of iodine is also very questionable, unless it be given, as indeed it generally is, with a view to act on a scrofulous constitution: by improving the general health it certainly will act indirectly on the eyes. The sulphate of quinine is also only useful when the patient is weak and feeble, and can merely benefit the eyes by benefitting the constitution in general.

From the cursory survey we have taken of the various remedial agents which are employed in the general treatment of acute iritis, you will perceive that, with the exception of blood-letting, we have but very little to boast of; that there is indeed no other general remedy on which any dependence can be placed. Snch a conclusion is not very flattering to our vanity, but I do not hesitate to make it known to you, as it is the result of my lengthened experience of the disease. It is, in my opinion, much preferable that we should be aware of our ignorance, on a subject with which we really are not acquainted, than that we should delude ourselves with the idea that we are perfectly conversant with it, as in the first case we continue our researches, whereas in the second we remain inactive.

There are a certain number of remedies, such as blisters, setons, moxas, &c., which partially deserve the name of general agents, and which ought, therefore, to be studied before we begin to examine the local treatment of the disease. Applied at the nape of the neck, and in the immediate vicinity of the head, blisters do not appear to exercise much influence over the progress of acute iritis, and as they may act on the lymphatic system, I think it is better not to employ them in those regions. In other parts of the body they may be beneficial; I occasionally apply them on the thighs, or on the calf of the legs, keeping them open for some time. I have in several instances surrounded the neck or the two arms of a patient with a large blister, but have never found the iritis to be sensibly modified by such a measure. In some cases of acute iritis I have applied blisters over the orbit, and this practice has been attended with so much benefit to the patients with whom I adopted it, that I feel inclined to give it a further trial. I have, as you all know, often followed this plan of treatment in the various inflammatory affections of the eye, and have never found reason to repent having so done.

Some authors have spoken of a remedy similar in its mode of action to cantharides plaster, as if the effects it produces were quite miraculous. A piece of linen is steeped in an effusion of mezereon root in strong acetic acid, and then applied to the skin, on which it produces vesication, exactly in the same manner as boiling water, or an ordinary blister. I cannot, however, understand why, the effects of these agents being identical, one should be so much more efficacious than the other.

Setons and moxas are also used in the treatment of iritis. They may be useful, but more so in the chronic than in the acute stage of the disease.—*Lond. Med. Gaz.*

---

*Statistics of Tracheomoty.*—Since the introduction of tracheotomy in croupal affections into France, there has, doubtless, been reason to deplore a great number of failures; it may be presumed, however, that one of the chief reasons of its insufficiency depends on the delay, and the obstacles that are generally thrown in the way of its performance. The following are the results which the different most celebrated operators have, by their own declarations, in a late discussion at the Royal Academy of Medicine in Paris, obtained.

| | Operations. | Cures. | Deaths. |
|---|---|---|---|
| M. Amussat - | 6 | 0 | 6 |
| Baudelocque - | 15 | 0 | 15 |
| Blandin - | 5 | 0 | 5 |
| Brettonneau - | 18 | 4 | 14 |
| Gerdy - | 6 | 4 | 2 |
| Roux - | 4 | 0 | 4 |
| Trosseau - | 80 | 20 | 60 |
| Velpeau - | 6 | 0 | 6 |
| | 150 | 28 | 112 |

So that, of 140 patients operated on 28 only have been cured, and 112 have died.—*Journal des Connais. Med. from Brit and. For. Med. Rev.*

---

*Ergot of Rye—its effects on the Fœtus.*

Mr. Proctor, in reference to the effect of ergot of rye on fœtal life, said that a friend of his, in extensive midwifery practice in the country, had, from considerable observation, come to the conclusion that it did effect the life of the fœtus. He, Mr. Proctor, thought this conclusion to be well founded. When labour was terminated by this agent, the action was unnatural, the pain was continuous instead of intermittent, and consequently violent. He had been alarmed in several cases, at the effect of the ergot of rye on the mother. He had seen it in one instance produce delirium and vertigo, and in another umbilical hernia. His friend in the country, to whom he had alluded, instead of taking ergot of rye in his pocket when he went to a labour, had now substituted tea and sugar, and this was acting upon the safe side.

Dr. Bennett inquired in what way it was supposed the ergot acted injuriously to the infant; was it by producing aplopexy in the mother, or by acting directly on fœtal life?

Mr. Statham had administered the ergot of rye in many cases, and he had come to the conclusion that it was injurious to fœtal life. He had at first thought its use admissible in all cases in which the os uteri was dilated to any extent. He now never gave it except when the child's head was in the pelvis, and then he had no doubt of its being advantageous, if the os uteri were flaccid. He considered that the ergot acted injuriously, by producing pressure upon the umbilical chord, and stopping the circulation.—*Proceedings of London Medical Society.—Lancet.*

